# Fitness costs in clothianidin-resistant population of the melon aphid, *Aphis gossypii*

**DOI:** 10.1371/journal.pone.0238707

**Published:** 2020-09-14

**Authors:** Farman Ullah, Hina Gul, Kaleem Tariq, Nicolas Desneux, Xiwu Gao, Dunlun Song

**Affiliations:** 1 Department of Entomology, China Agricultural University, Beijing, China; 2 Department of Agriculture Entomology, Abdul Wali Khan University Mardan, Khyber Pakhtunkhwa, Pakistan; 3 Entomology and Nematology Department, Steinmetz Hall, University of Florida, Gainesville, Florida, United States of America; 4 USDA/ARS, Center for Medical, Agricultural and Veterinary Entomology, Gainesville, Florida, United States of America; 5 Université Côte d’Azur, INRAE, CNRS, UMR ISA, Nice, France; Al-Azhar University, EGYPT

## Abstract

Clothianidin is a second-generation neonicotinoid insecticide, widely used against sap-sucking insect pest including melon aphid, *Aphis gossypii* Glover (Hemiptera: Aphididae). This pest causes severe economic damage to Cucurbitaceae plants worldwide. In this study, we investigated clothianidin resistance development under continuous selection pressure. Moreover, the age-stage, two-sex life table approach was used to evaluate the impact of clothianidin resistance on the fitness of *A*. *gossypii*. A clothianidin resistant strain (CT-R) with a 23.17-fold resistance level was developed from a susceptible strain (CT-S) after continuous selection for 24 generations. Life table results showed a significant reduction in the relative fitness (0.847) of CT-R strain compared to the CT-S strain of *A*. *gossypii*. The developmental duration, oviposition days, total pre-oviposition period (TPOP), longevity, and fecundity of CT-R strain were found to be significantly lower when compared to CT-S strain. The demographic parameters, including the intrinsic rate of increase (*r*), finite rate of increase (*λ*), net reproductive rate (*R*_0_), and mean generation time (*T*) were also significantly decreased in CT-R strain compared to the CT-S strain. Both the reproductive and survival rates were affected by clothianidin resistance in CT-R strain compared with the CT-S strain of *A*. *gossypii*. Overall, our results demonstrate that in-depth knowledge about the trade-off at play between resistance degree and fitness cost might be useful to design resistance management strategies against *A*. *gossypii*.

## Introduction

The melon aphid, *Aphis gossypii* Glover (Hemiptera: Aphididae), is an economically important insect pest, affecting Cucurbitaceae plants worldwide [1]. *Aphis gossypii* causes severe economic losses by direct feeding i.e., deforming the young leaves and twigs and indirectly by transmitting several plant viruses [1, 2]. Various control methods were used [3–5], but still, synthetic chemical insecticides are considered crucial to combat aphids [6–8], despite negative effects on beneficial insects [9–14]. However, the indiscriminate applications of chemical insecticides cause resistance development in insect pests, including *A*. *gossypii* [15–20].

Neonicotinoid insecticides, including clothianidin, have been widely used against several insect pests [8, 19, 21–24]. Neonicotinoids act as an agonist of nicotinic acetylcholine receptors (nAChRs), causing nerve stimulation, paralysis, and death [25–27]. Owing to stomach and contact insecticidal activity, neonicotinoids are considered one of the most widely used insecticide group against several insect pests including aphids, whiteflies, jassids, leafminers, thrips, monarch butterfly and many species of beetles (Coleoptera: Curculionidae) [8, 15, 28–36]. Clothianidin is a second-generation neonicotinoid insecticide broadly used for controlling aphids [8, 26, 31]. The resistant populations of *A*. *gossypii* suffer adverse effects on life history traits in the absence of the insecticide [8]. The LC_15_ of clothianidin affects the longevity, fecundity, and demographic parameters (*R*_0_ and *GRR*) of *A*. *gossypii* [8]. Acetamiprid is also a neonicotinoid insecticide widely used against several sap-sucking insect pests, including *A*. *gossypii*. [21, 37–40]. Both the longevity and fertility of *A*. *gossypii* were decreased when exposed to the LC_5_ and LC_15_ concentrations of acetamiprid [21].

Fitness cost affects insecticide resistance evolution and the rate of resistance increase in insects [41, 42]. Resistance costs energy and that may influence fitness in the absence of the insecticide stressor [42]. Prior studies showed evidence about the fitness costs associated with insecticide resistance in several insects including *Bradysia odoriphaga* Yang and Zhang (Diptera: Sciaridae), *Thrips hawaiiensis* Morgan (Thysanoptera: Thripidae), *Plutella xylostella*, Linnaeus (Lepidoptera: Plutellidae), *Nilaparvata lugens* Stål (Hemiptera: Delphacidae) *N*. *lugens* and *Musca domestica* Linnaeus (Diptera: Muscidae). Ma et al. reported fitness costs of 0.917 in the sulfoxaflor resistant population of *A*. *gossypii* compared to the susceptible strain [43]. The relative fitness had also been decreased up to 0.83 in the sulfoxaflor resistant strain of *Myzus persicae* Sulzer (Homoptera: Aphididae) [44]. The life-history traits of *B*. *odoriphaga* have significantly been affected in clothianidin and chlorfenapyr resistant populations compared to the susceptible strain [15, 45]. Several studies have reported the development of insecticide resistance accompanied by fitness costs in different insect pests [15, 46–49].

To our knowledge, no study to date has examined selection-induced clothianidin resistance development accompanied with fitness costs in *A*. *gossypii*. The overall goal of this work was to analyze the risk of clothianidin resistance in *A*. *gossypii* under continuous selection pressure. To examine the impact of clothianidin resistance on fitness, we used the age-stage two-sex life table approach to accurately quantify the life history traits of resistant (CT-R) and susceptible (CT-S) strains of *A*. *gossypii*. This gives an in-depth knowledge about the optimal application of clothianidin insecticides against *A*. *gossypii*.

## Materials and methods

### Insects and insecticide

*Aphis gossypii* were initially collected from melon plants at Weifang City, Shandong Province, China. The population was maintained under standard laboratory conditions (25 ± 1°C; 75% RH; 16:8 L: D) at China Agricultural University using insecticide-free cucumber plants. Technical grade clothianidin (95% of the active ingredient) was purchased from Bayer CropScience Co. Ltd (Monheim, Germany). Triton X-100 was obtained from Sigma-Aldrich Co. Saint Louis, USA.

### Ethics approval

No ethics approval was required for this research.

### Toxicity of clothianidin against *A*. *gossypii*

The bioassays of clothianidin were conducted under laboratory conditions using the leaf-dipping procedure. The stock solution of clothianidin was prepared in acetone, and further dilution was set up in distilled water containing 0.05% (v/v) Triton X-100. Cucumber leaf discs were dipped in the required diluted concentrations of clothianidin or in 0.05% (v/v) Triton X-100 water as a control for 15 s. The treated discs were allowed to dry on the disposable transparent plastic gloves and then placed the adaxial side of leaf discs on 2% (w/v) agar bed (2 ml) in a 12-well cell culture plate. Twenty adult melon aphids were placed on each leaf disc, and Chinese art paper (Xuan rice paper) was used to cover the plate to prevent the aphid’s escape. There were three leaf disks for each concentration, and the entire experiment was repeated three times for a total of 180 aphids tested at each concentration. The mortality was recorded at 72 h after treatment. The LC_50_ values of clothianidin were calculated by probit analysis using POLO Plus 2.0 statistical software.

### Establishing the resistant colony

The resistant strain of clothianidin (CT-R) was established from an originally collected susceptible population of *A*. *gossypii* through continuous selection pressure for 24 generations. The acute toxicity of clothianidin was recorded for each generation. Based on the results of the bioassays of the parent aphids, the clothianidin concentrations were gradually increased throughout the selection experiment. The mortality rate was maintained at 60–80%. The resistance ratio (RR) was determined at each generation by dividing the LC_50_ of resistant strain to the LC_50_ of the susceptible strain of *A*. *gossypii*. The susceptible strain (CT-S) was maintained on cucumber plants without any pre- or post-exposure of clothianidin. Both strains were kept under standard laboratory conditions (25 ± 1 °C; 75% RH; 16:8 L: D) in the Department of Entomology, China Agricultural University, Beijing, China.

### Fitness comparisons

Fitness of the susceptible and resistant strains of *A*. *gossypii* was compared using age-stage, two-sex life table approach. About 500 apterous adults were inoculated to insecticide-free cucumber seedlings. After 24 h, ninety newly born nymphs of *A*. *gossypii* were collected from both susceptible and resistant populations. Both strains were transferred to insecticide-free cucumber seedlings and were maintained separately under laboratory conditions. Each individual aphid growing on one insecticide-free cucumber seedling was considered as a single replicate [8, 19]. Nymphs from both populations were observed individually and we recorded development duration, mortality, longevity, and fecundity. The life table data of susceptible (CT-S) and resistant (CT-R) strains of *A*. *gossypii* were subjected to the TWOSEX-MSChart computer program [50] to analyze the population parameters including age-stage specific survival rates (*s*_*xj*_), age-specific survival rate (*l*_*x*_), age-specific fecundity (*m*_*x*_), age-specific maternity (*l*_*x*_*m*_*x*_), age-stage specific life expectancy (*e*_*xj*_) and age-stage reproductive value (*v*_*xj*_) following age-stage two-sex life table procedure [51]. The *s*_*xj*_ shows the probability that a newly born nymph will survive to age *x* and stage *j*. The *l*_*x*_ represents a simplified form of the survival history and the probability that a newly-born nymph will survive to age *x*. The *m*_*x*_ indicates the age-specific fecundity, while the *l*_*x*_*m*_*x*_ shows age-specific maturity. The *v*_*xj*_ depicts the devotion to future offspring for *A*. *gossypii* individuals of at age *x* and stage *j*. The *e*_*xj*_ describes the expected duration of time an individual of age *x* at stage *j* that will survive after the age *x*. The newly born nymphs produced by females during the reproductive period were counted and removed daily. Fresh cucumber seedlings were replaced after 5 days without any insecticide exposure throughout the experiment. The aphids were individually transferred to new seedlings using a soft brush.

### Statistical analysis

The age-stage two-sex life table procedure was applied to analyze the developmental duration, adult longevity, and fecundity for all individual aphids using the TWOSEX-MSChart computer program [50–53]. The population parameters including the intrinsic rate of increase (*r*), finite rate of increase (*λ*), net reproductive rate (*R*_*0*_), mean generation time (*T*), gross reproduction rate (*GRR*), adult pre-oviposition period (APOP), total pre-oviposition period (TPOP), oviposition days (Od), age-stage specific survival rates (*s*_*xj*_), age-specific survival rate (*l*_*x*_), age-specific fecundity (*m*_*x*_), age-specific maternity (*l*_*x*_*m*_*x*_), age-stage specific life expectancy (*e*_*xj*_) and age-stage reproductive value (*v*_*xj*_) were investigated following Chi and Liu [52] and Chi [53] using TWOSEX-MSChart computer program [50]. The means and standard errors of the population parameters between CT-S and CT-R strains were analyzed using paired bootstrap tests via 100,000 bootstrap replicates [51, 54, 55]. All figures were constructed using SigmaPlot 12.0 (Systat Software Inc., San Jose, CA, USA).

## Results

### Clothianidin resistance development

The clothianidin resistant strain (CT-R) was established from the susceptible strain (CT-S) through continuous exposure with clothianidin for 24 generations under controlled conditions (Table 1).

**Table 1:** The resistance level of *Aphis gossypii* to clothianidin.

Generations	LC_50_ (95%CI)^a^ mg L^-1^	Slope ± SE^b^	*χ*^2^	*P*-value	RR^c^
F0	0.38 (0.28–0.49)	1.95±0.24	16.50	0.223	-
F2	0.52 (0.41–0.66)	1.62 ± 0.21	3.02	0.998	1.38
F4	0.86 (0.67–1.16)	1.55 ± 0.22	2.40	0.999	2.27
F6	1.29 (1.00–1.86)	1.61 ± 0.23	2.49	0.999	3.43
F8	1.55 (1.27–1.92)	2.02 ± 0.24	3.51	0.995	4.09
F10	2.23 (1.73–3.15)	1.55 ± 0.22	2.92	0.998	5.91
F12	2.79 (2.33–3.38)	2.25 ± 0.28	4.12	0.990	7.39
F14	3.52 (2.88–4.50)	1.94 ± 0.25	2.62	0.999	9.31
F16	4.47 (3.83–5.17)	2.94 ± 0.45	7.62	0.868	11.83
F18	5.56 (4.72–6.79)	2.38 ± 0.34	3.22	0.997	14.70
F20	6.78 (5.76–8.54)	2.63 ± 0.42	2.46	0.999	17.93
F22	7.72 (6.42–9.26)	2.15 ± 0.23	2.56	0.999	20.43
F24	8.76 (6.95–11.06)	1.65 ± 0.21	3.25	0.997	23.17

Number of larvae exposed in bioassay, including control = 360; df = 13.

^a^ 95% confidence intervals.

^b^ Standard error.

^c^ RR, resistance ratio, determined as (LC_50_ of resistant strain/LC_50_ of susceptible strain).

The LC_50_ value of the CT-S was 0.38 mg L^−1^. In the first 10 generations (F_2_-F_10_) of the CT-R population, the LC_50_ values were slowly increased from 0.52 mg L^−1^ to 2.23 mg L^−1^. However, these values were steeply increased in the following generations (F_12_-F_24_) with LC_50_ values 2.79, 3.52, 4.47, 5.56, 6.78, 7.72, and 8.76 mg L^−1^, respectively. After the selection for 24 generations, the resistance ratio was increased (23.17-fold) compared to the CT-S strain (Table 1).

### Impact of clothianidin resistance on the life history traits of *A*. *gossypii*

The life-history traits, including developmental time, longevity, fecundity, and oviposition days between clothianidin resistant (CT-R) and susceptible strains (CT-S) of *A*. *gossypii* are presented in Table 2. The mean developmental durations of 1^st^ instar, 3^rd^ instar, and 4^th^ instar nymph of CT-R strain was significantly shorter than that of the CT-S aphids (*P* <0.001). The pre-adult period, adult duration, and total pre-oviposition period (TPOP) were significantly shorter in CT-R strain compared to the CT-S strain (Table 2). No significant differences were observed for the adult pre-oviposition period (APOP) between both strains of *A*. *gossypii*. The oviposition days, total longevity and fecundity were significantly lower in CT-R strain of *A*. *gossypii* (*P* <0.05).

**Table 2:** Mean (± SE) life history parameters of the susceptible (CT-S) and resistant (CT-R) strains of *Aphis gossypii*.

Stages	Susceptible strain (CT-S)		Resistant strain (CT-R)		95% CI ^c^	*P*-value
	n ^a^	Mean ± SE^b^	n ^a^	Mean ± SE^b^		
First instar nymph (days)	90	1.99 ± 0.07	90	1.61 ± 0.08	(0.170, 0.585)*	<0.001
Second instar nymph (days)	89	1.63 ± 0.08	89	1.51 ± 0.07	-0.091, 0.339)	0.258
Third instar nymph (days)	87	1.72 ± 0.09	85	1.33 ± 0.07	(0.173, 0.616)*	0.001
Fourth instar nymph (days)	86	1.92 ± 0.06	83	1.54 ± 0.09	(0.170, 0.583)*	<0.001
Pre-adult (days)	86	7.28 ± 0.10	83	6.01 ± 0.08	(1.014, 1.521)*	<0.001
Adult (days)	86	19.16 ± 0.48	83	16.17 ± 0.57	(1.528, 4.461)*	<0.001
APOP (days)	86	0.23 ± 0.05	83	0.25 ± 0.06	(-0.178, 0.137)	0.798
TPOP (days)	86	7.51 ± 0.11	83	6.27 ± 0.10	(0.950, 1.544)*	<0.001
Oviposition days	86	19.41 ± 1.16	83	14.41 ± 0.51	(2.459, 7.536) *	<0.001
Total longevity (days)	90	25.46 ± 0.68	90	20.77 ± 0.75	(2.692, 6.686)*	<0.001
Fecundity (offspring/individual)	86	41.38 ± 0.96	83	36.35 ± 1.15	(2.081, 7.988)*	0.001

^a^ Number of subjects.

^b^ Standard errors (SE) were estimated using the bootstrap technique with 100,000 re-samplings.

^c^ Difference between strains were compared with paired bootstrap test. If the CI includes 0, there is no difference at 5% level.

* Significant differences between resistant strain (CT-R) and susceptible strain (CT-S) at *P* = 0.05 level, paired bootstrap test using TWOSEX MS chart program.

The demographic traits (*r*, *λ*, *R*_0_, *T*, and *GRR*) of CT-R and CT-S strains of *A*. *gossypii* were evaluated by a paired bootstrap technique based on the life table (Table 3). When compared to CT-S strain, the *r* and *λ* of CT-R strain were significantly increased (*P* <0.001). While the *R*_*0*_, *T*, and *GRR* in CT-R strain were markedly decreased as compared to the CT-S strain of *A*. *gossypii* (Table 3). In the absence of insecticide exposure, the overall fitness of clothianidin-resistant strain (CT-R) of *A*. *gossypii* was 0.847 as compared to the susceptible strain (CT-S).

**Table 3:** Mean (± SE) demographic parameters of the susceptible (CT-S) and resistant (CT-R) strains of *Aphis gossypii*.

Population parameters ^a^	(Mean ± SE ^b^)		95% CI ^c^	*P*-value
	Susceptible strain (CT-S)	Resistant strain (CT-R)		
*r* (d^−1^)	0.2821 ± 0.0040	0.3165 ± 0.0055	(0.0208, 0.0479)*	<0.001
*λ* (d^−1^)	1.3259 ± 0.0054	1.3724 ± 0.0076	(0.0280, 0.0648)*	<0.001
*R*_*0*_ (offspring/individual)	39.5478 ± 1.2800	33.5286 ± 1.4674	(2.2011, 9.8371)*	0.002
*T* (days)	13.0336 ± 0.1751	11.0947 ± 0.1839	(1.4404, 2.4374)*	<0.001
*GRR* (offspring/individual)	52.0845 ± 1.0530	48.9539 ± 1.2530	(-0.0738, 6.3351)	0.056
*R*_*f*_ ^d^	-	0.847	-	-

^a^ *r*: intrinsic rate of increase, *λ*: finite rate of increase, *R*_0_: net reproductive rate, *T*: mean generation time, *GRR*: gross reproductive rate.

^b^ Standard errors (SE) were estimated using the bootstrap technique with 100,000 re-samplings.

^c^ Difference between strains were compared with paired bootstrap test. If the CI includes 0, there is no difference at 5% level.

^d^ *R*_*f*_ = *R*_*0*_ of the resistant strain (CT-R)/ *R*_*0*_ of the susceptible strain (CT-S).

* Significant differences between resistant strain (CT-R) and susceptible strain (CT-S) at *P* = 0.05 level, paired bootstrap test using TWOSEX MS chart program.

**Fig 1:**
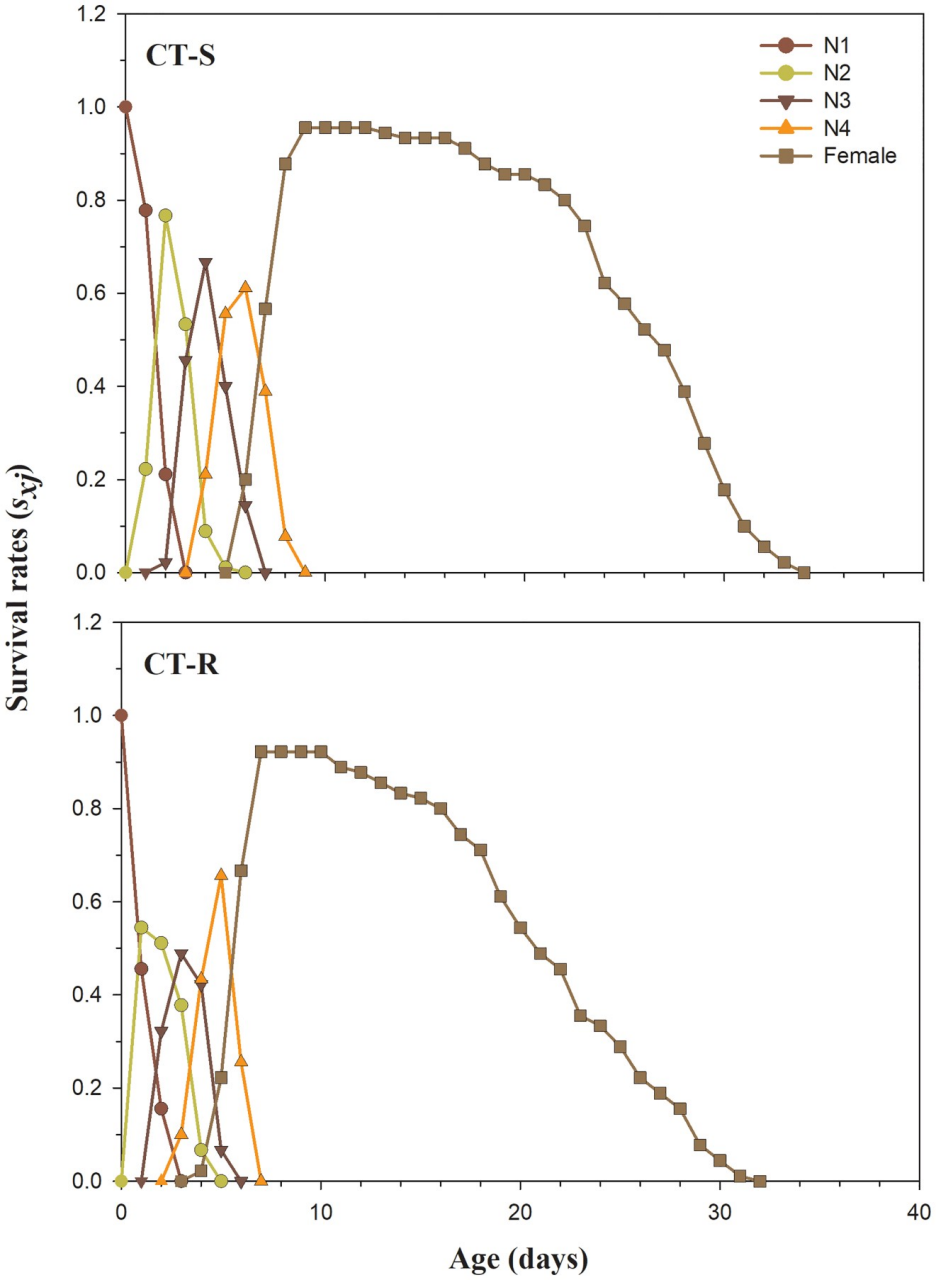
Age-stage specific survival rate (*s*_*xj*_) in susceptible (CT-S) and resistant (CT-R) strains of *A*. *gossypii*.

The *s*_*xj*_ shows the probability of neonate nymph that will survive to age x and stage j. The overlaps among different stages occurred due to the stage differentiations between CT-R and CT-S individuals (Fig 1). The *s*_*xj*_ curves show apparent differences, with a lower survival rate of nymphal and adult female stages in CT-R strain compared to the CT-S strain. The adult female survival rate of CT-R strain started to decline on 10^th^ day, while decline occurred on 12th day in CT-S strain (Fig 1).

**Fig 2:**
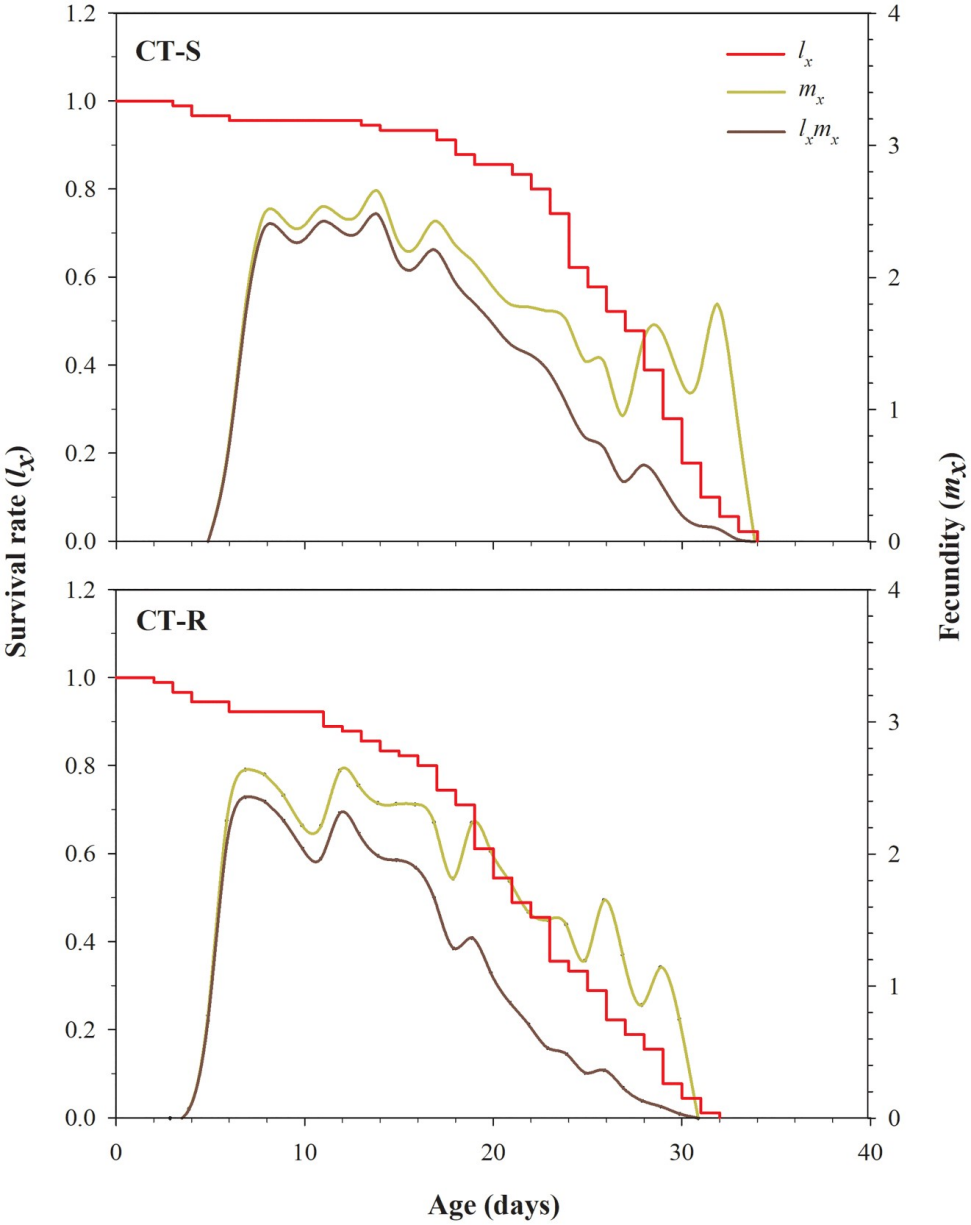
Age-specific survival rate (*l*_*x*_), age-specific fecundity (*m*_*x*_) and age-specific maternity (*l*_*x*_*m*_*x*_) in susceptible (CT-S) and resistant (CT-R) strains of *A*. *gossypii*.

**Fig 3:**
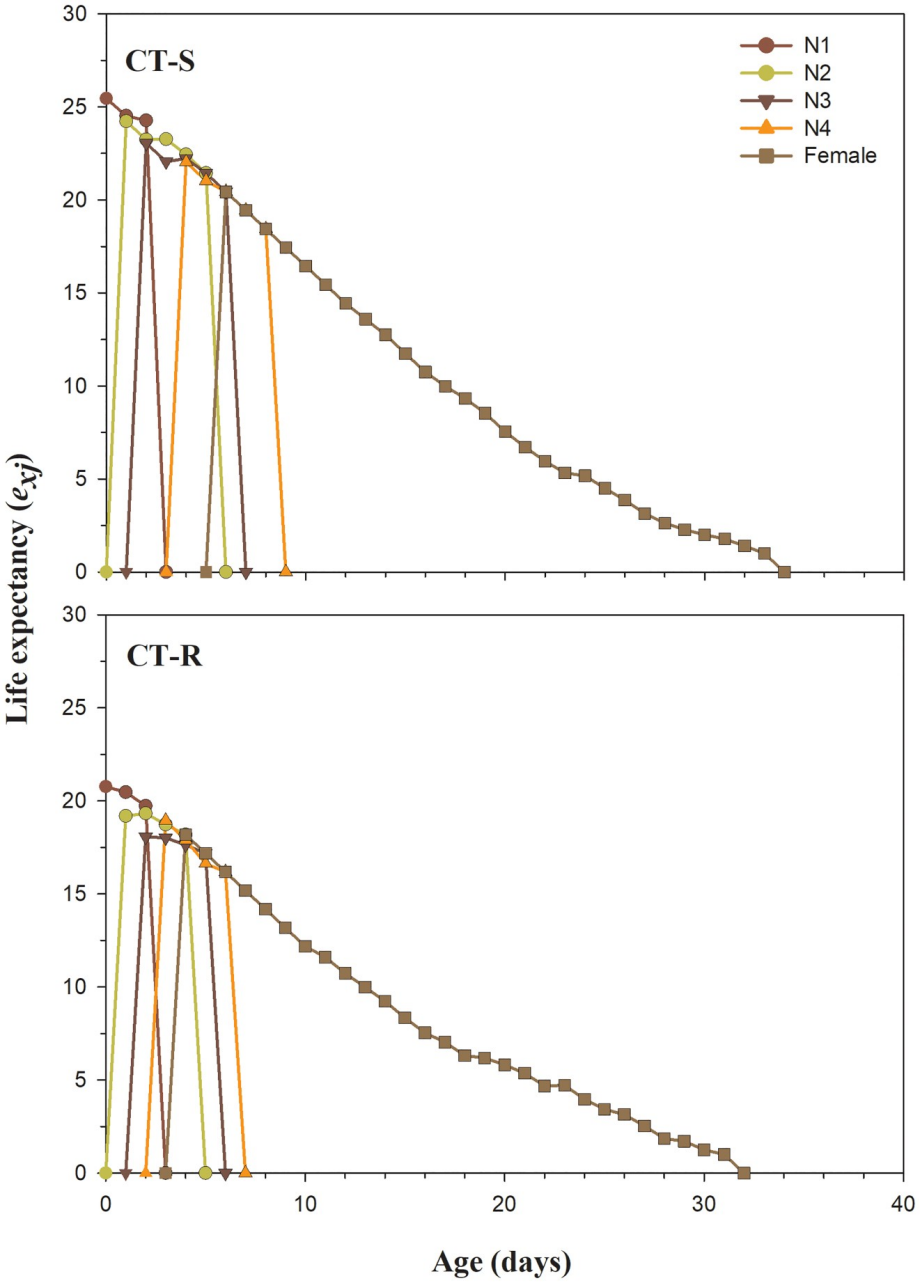
Age-stage specific life expectancy (*e*_*xj*_) in susceptible (CT-S) and resistant (CT-R) strains of *A*. *gossypii*.

**Fig 4:**
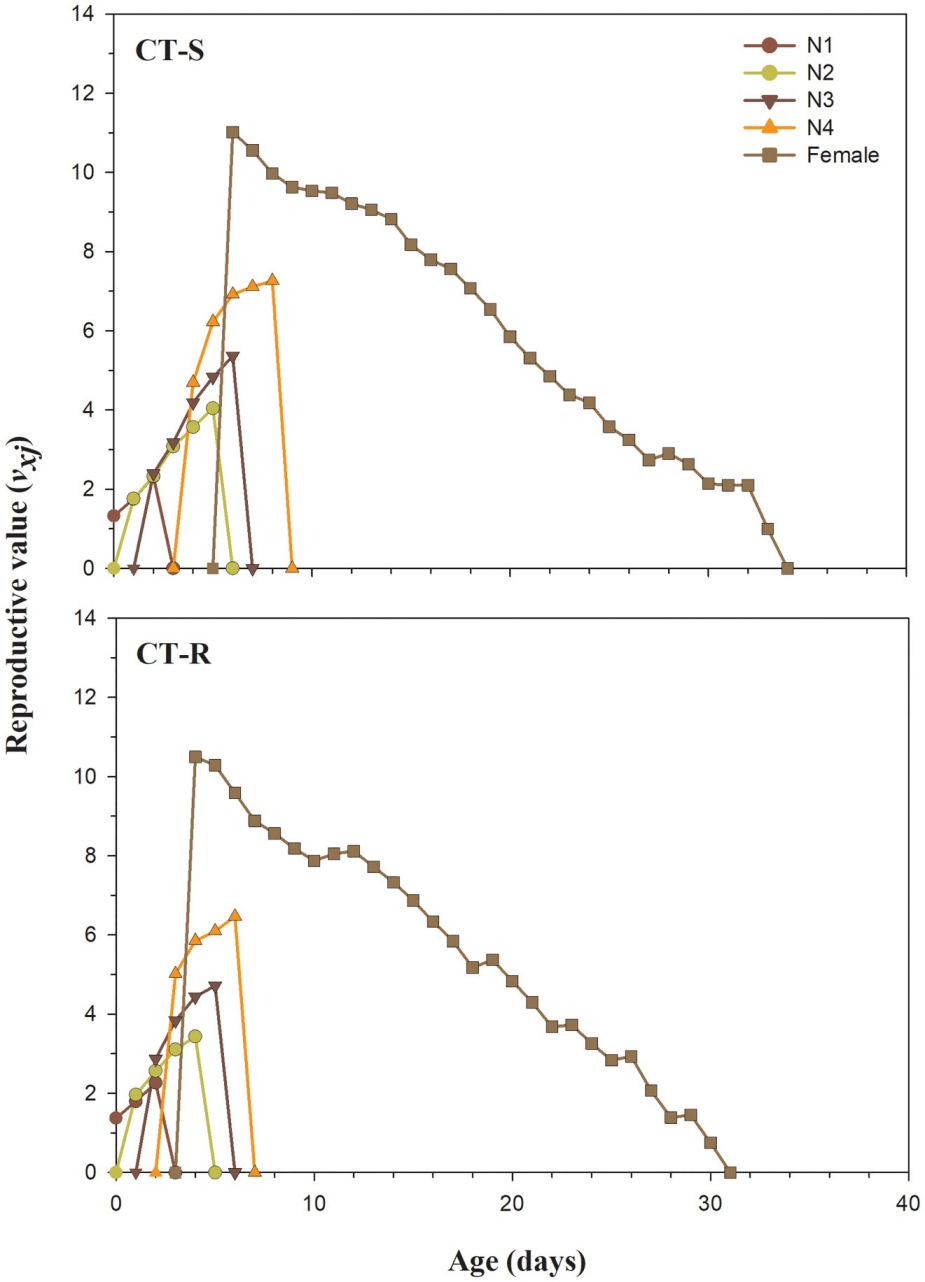
Age-stage reproductive value (*v*_*xj*_) in susceptible (CT-S) and resistant (CT-R) strains of *A*. *gossypii*.

The *l*_*x*_, *m*_*x*_ and *l*_*x*_*m*_*x*_ differences among CT-R and CT-S strains are presented in Fig 2. The *l*_*x*_ curves show a lower survival rate of CT-R strain at the age of 12–32 days compared to the CT-S strain of *A*. *gossypii* (Fig 2). The maximal survival duration of CT-R strain was 32 days, which was lower than that of the CT-S strain (34 days). The *m*_*x*_ and *l*_*x*_*m*_*x*_ values in CT-R strain was decreased after 18^th^ day, which shows lower fecundity as compared to the CT-S strain (Fig 2). In the first 12 days, values of *m*_*x*_ and *l*_*x*_*m*_*x*_ for CT-R strain were higher as compared to CT-S strain; however, after that, this trend has been reversed, and CT-R strain displayed lower fecundity as compared to the CT-S strain (Fig 2). The *e*_*xj*_ curves in CT-R strain show the shorter survival expectancy of the developmental as well as adult stage compared to the CT-S strain of *A*. *gossypii* (Fig 3). The *v*_*xj*_ shows the devotion of individuals of age x and stage j towards future offspring (Fig 4). The pattern of *v*_*xj*_ was recorded lower for the CT-R strain as compared to the CT-S of *A*. *gossypii* (Fig 4).

## Discussion

Chemical applications are still crucial for the control of *A*. *gossypii* in China [6, 56–58]. Clothianidin belongs to second-generation neonicotinoid insecticide that acts as an agonist of the nicotinic acetylcholine receptors (nAChRs) [27]. Due to excellent insecticidal activity, clothianidin has been broadly used against many insect pests including hemipterans and many species of beetles [8, 28–33]. However, the insecticidal actions have been dramatically affected by the development of insecticide resistance [15, 43, 45]. There have been numerous studies to investigate the development of insecticide resistance against various pests such as Chive maggot, Colorado potato beetle, small brown plant hopper, western flower thrips, peach aphid and tobacco aphid [15, 45, 59–62]. The resistance development accompanied by fitness costs significantly affect the evolution of insecticide resistance [43, 45]. To our knowledge, no previous research has investigated the development of clothianidin resistance and associated fitness costs in the melon aphid, *A*. *gossypii*. Therefore, an in-depth study about the resistance development and fitness costs of a resistant population of *A*. *gossypii* could be crucial for an effective control measure against this insect pest.

We showed that *A*. *gossypii* developed 23.17-fold clothianidin resistance following 24 generations of exposure to increasing concentrations of clothianidin. Recent studies by Ullah et al. and Gul et al. concluded 43.32- and 76-fold resistance in *B*. *odoriphaga* following 10 consecutive generations selection to chlorfenapyr and clothianidin, respectively [15, 45]. Beet armyworm, *Spodoptera exigua* Hübner (Lepidoptera: Noctuidae) showed 69.76- and 113.29-fold resistance against deltamethrin and gossypol, respectively following 10 generations selections [63]. There have been numerous studies to investigate selection-induced resistance in many insect and insecticide combinations [64–68]. The resistance ratio was lower in CT-R strain of *A*. *gossypii*, as compared to other insects and insecticides. The difference might be due to differences in the field collected material, their prior exposure to insecticides, and the length of time the colony was maintained insecticide-free in the laboratory. The resistance ratio might be higher if the population exposed to clothianidin for more generations, which is a future prospect. Also, different insect species show different responses to insecticides.

Fitness costs associated with resistance have been broadly studied in several insect species including *B*. *odoriphaga*, *T*. *hawaiiensis*, *P*. *xylostella*, *N*. *lugens* and *M*. *domestica* [15, 41, 45, 69–71]. In the current study, two strains with similar genetic backgrounds have been used to accurately assess the resistance-linked fitness costs [45]. The results have shown the shorter developmental duration of 1^st^ instar, 3^rd^ instar, and 4^th^ instar nymphs of CT-R strain as compared to the CT-S strain of *A*. *gossypii*. The pre-adult period of CT-R was also shorter than that of the CT-S aphids. This shows that the clothianidin resistance development could facilitate the nymphal growth in *A*. *gossypii*. As has been previously reported in the literature, suggesting the decreased developmental durations of nymphal stages and pre-adult period in sulfoxaflor-resistant *A*. *gossypii* [43]. Several studies have reported similar phenomena in *M*. *persicae* and *A*. *gossypii* resistant populations [44, 72]. The developmental duration was also decreased in deltamethrin and gossypol resistant strains of *S*. *exigua* compared to susceptible strain [63]. In contrast, others have shown increased developmental durations in the resistant insect pests compared to the susceptible strain [71, 73]. For example, the developmental period of *B*. *odoriphaga* significantly enhanced in clothianidin resistant strain [15].

In current study, the adult longevity, TPOP, oviposition period, total longevity, and fecundity were decreased significantly in CT-R strain. From the results, it is clear that the clothianidin resistance developed at the cost of the reduced fecundity. Overall these findings are in accordance with findings reported by Ullah et al. and Gul et al. that development of insecticide resistance affects the life-history traits, including fecundity and longevity of the resistant strain [15, 45]. The longevity and fecundity were decreased significantly in deltamethrin and gossypol resistant strains of *S*. *exigua* [63]. The shorter longevity (9.55%) and fecundity (15%) were also observed in the resistant strain of *M*. *persicae* [44]. Resistance-induced fitness costs have been reported in several other insect pests [70, 74–78].

The demographic traits can explain the growth potential of insect pest populations [79, 80]. The *r* and *λ* were significantly increased in CT-R compared to the CT-S. However, the *R*_0_ and *T* were decreased in clothianidin resistant strain of *A*. *gossypii*. The findings are directly in line with previous findings of Ma et al. showing that the *r* and *λ* were increased, while *R*_0_ and *T* were decreased in sulfoxaflor-resistant *A*. *gossypii* [43]. The *R*_0_ was lowered in the laboratory selected resistant strains of *M*. *persicae* and *B*. *odoriphaga* [15, 44, 45]. The demographic traits, including *r*, *λ*, *R*_*0*_, and *T* were also affected by clothianidin and gossypol resistance in *S*. *exigua* [63].

The *s*_*xj*_, *l*_*x*_, *m*_*x*_, *l*_*x*_*m*_*x*_, *e*_*xj*_ and *v*_*xj*_ were significantly decreased in clothianidin resistant strain of *A*. *gossypii*. Our findings are consistent with Ullah et al. and Gul et al. showing similar results in clothianidin and chlorfenapyr resistant strain of *B*. *odoriphaga* [15, 45]. Many prior reports have showed similar effects on several insect and insecticide combinations including sulfoxaflor-resistant *A*. *gossypii* [43], imidacloprid-resistant *S*. *litura* [75], deltamethrin and indoxacarb-resistant *Heliothis virescens* Fabricius (Lepidoptera:Noctuidae) [77] and spinosad-resistant *P*. *xylostella* [81]. From the results, it is clear that *A*. *gossypii* has the potential to develop resistance against widely used clothianidin insecticide. Our study also provides a comprehensive understanding of the fitness costs in CT-R as compared to CT-S.

## Conclusion

Overall, our results show clothianidin resistance development (23.17-fold) in *A*. *gossypii* under continuous selection over 24 generations. Moreover, there are fitness costs in the resistant population, owing to the selection of resistance to clothianidin in *A*. *gossypii*. These findings will be useful for understanding clothianidin resistance and associated fitness costs in *A*. *gossypii*. However, future research on the underlying molecular mechanisms might extend the explanations of clothianidin resistance in *A*. *gossypii*.

## Supporting information

S1 Table Life table of susceptible strain (CT-S) of *A*. *gossypii*. (XLSX)

S2 Table Life table of clothianidin-resistant strain (CT-R) of *A*. *gossypii*. (XLSX)
